# Enhanced Feedback Inhibition Due to Increased Recruitment of Somatostatin-Expressing Interneurons and Enhanced Cortical Recurrent Excitation in a Genetic Mouse Model of Migraine

**DOI:** 10.1523/JNEUROSCI.0228-22.2022

**Published:** 2022-08-24

**Authors:** Ivan Marchionni, Nadia Pilati, Angelo Forli, Michele Sessolo, Angelita Tottene, Daniela Pietrobon

**Affiliations:** ^1^Department of Biomedical Sciences, University of Padova, Padova 35131, Italy; ^2^Padova Neuroscience Center, University of Padova, and National Research Council Institute of Neuroscience, Padova 35131, Italy; ^3^Autifony Srl, Istituto di Ricerca Pediatrica Citta' della Speranza, Padova 35127, Italy

**Keywords:** calcium channel, feedback inhibition microcircuit, migraine, somatosensory cortex, somatostatin interneuron, synaptic transmission

## Abstract

Migraine is a complex brain disorder, characterized by attacks of unilateral headache and global dysfunction in multisensory information processing, whose underlying cellular and circuit mechanisms remain unknown. The finding of enhanced excitatory, but unaltered inhibitory, neurotransmission at cortical synapses between pyramidal cells (PCs) and fast-spiking interneurons (FS INs) in mouse models of familial hemiplegic migraine (FHM) suggested the hypothesis that dysregulation of the excitatory-inhibitory (E/I) balance in specific circuits is a key pathogenic mechanism. Here, we investigated the cortical layer 2/3 (L2/3) feedback inhibition microcircuit involving somatostatin-expressing (SOM) INs in FHM1 mice of both sexes carrying a gain-of-function mutation in Ca_V_2.1. Unitary inhibitory neurotransmission at SOM IN-PC synapses was unaltered while excitatory neurotransmission at both PC-SOM IN and PC-PC synapses was enhanced, because of increased probability of glutamate release, in FHM1 mice. Short-term synaptic depression was enhanced at PC-PC synapses while short-term synaptic facilitation was unaltered at PC-SOM IN synapses during 25-Hz repetitive activity. The frequency-dependent disynaptic inhibition (FDDI) mediated by SOM INs was enhanced, lasted longer and required shorter high-frequency bursts to be initiated in FHM1 mice. These findings, together with previous evidence of enhanced disynaptic feedforward inhibition by FS INs, suggest that the increased inhibition may effectively counteract the increased recurrent excitation in FHM1 mice and may even prevail in certain conditions. Considering the involvement of SOM INs in γ oscillations, surround suppression and context-dependent sensory perception, the facilitated recruitment of SOM INs, together with the enhanced recurrent excitation, may contribute to dysfunctional sensory processing in FHM1 and possibly migraine.

**SIGNIFICANCE STATEMENT** Migraine is a complex brain disorder, characterized by attacks of unilateral headache and global dysfunction in multisensory information processing, whose underlying cellular and circuit mechanisms remain unknown, although dysregulation of the excitatory-inhibitory (E/I) balance in specific circuits could be a key pathogenic mechanism. Here, we provide insights into these mechanisms by investigating the cortical feedback inhibition microcircuit involving somatostatin-expressing interneurons (SOM INs) in a mouse model of a rare monogenic migraine. Despite unaltered inhibitory synaptic transmission, the disynaptic feedback inhibition mediated by SOM INs was enhanced in the migraine model because of enhanced recruitment of the INs. Recurrent cortical excitation was also enhanced. These alterations may contribute to context-dependent sensory processing dysfunctions in migraine.

## Introduction

Migraine is a common, disabling brain disorder, characterized by a global dysfunction in multisensory information processing and by attacks of typically throbbing, unilateral headache with certain associated features (Pietrobon and Moskowitz, 2013; Brennan and Pietrobon, 2018). In a third of patients, the headache is preceded by transient sensory disturbances (aura), whose neurophysiological correlate is cortical spreading depression (CSD), a cortical wave of nearly complete neuronal depolarization (Pietrobon and Moskowitz, 2014), which may trigger the headache mechanisms (Burstein et al., 2015; Filiz et al., 2019; Harriott et al., 2021). The neurobiological mechanisms of the primary brain dysfunctions underlying the onset of a migraine attack, susceptibility to CSD and the global dysfunction in multisensory information processing remain largely unknown. Familial hemiplegic migraine (FHM) is a rare monogenic subtype of migraine with aura caused by mutations in genes encoding either neuronal voltage-gated ion channels (Ca_V_2.1 in FHM1; Na_V_1.1 in FHM3) or the predominantly astrocytic α_2_ Na^+^, K^+^ ATPase (in FHM2; Pietrobon, 2007). Knock-in mouse models of FHM provide a unique experimental system to study the cellular and circuit mechanisms of the primary brain dysfunctions causing a migraine disorder (Pietrobon and Brennan, 2019).

FHM1 knock-in mice (FHM1 mice) carry gain-of-function mutations in the Ca_V_2.1 channel, which plays a dominant role in neurotransmitter release at many brain synapses (van den Maagdenberg et al., 2004, 2010; Pietrobon, 2013). FHM1 mice show unaltered cortical inhibitory neurotransmission at fast-spiking interneurons (FS INs) synapses and enhanced neurotransmission at several excitatory synapses, including the intracortical synapses between layer 2/3 (L2/3) pyramidal cells (PCs) and FS-INs and the thalamocortical synapses between thalamocortical neurons and either L4 principal neurons or FS INs (Tottene et al., 2009, 2019; Vecchia et al., 2014, 2015). FHM1 mice show a lower threshold for CSD induction, which is because of the enhanced cortical glutamatergic neurotransmission (van den Maagdenberg et al., 2004, 2010; Tottene et al., 2009). These findings suggested that impaired regulation of the excitatory-inhibitory (E/I) balance in specific neuronal circuits may be a key pathogenic mechanism in FHM and possibly idiopathic migraine (Vecchia and Pietrobon, 2012; Brennan and Pietrobon, 2018; Pietrobon, 2018). However, the specific neuronal circuits whose dysfunctional regulation may be responsible for the interictal alterations in sensory processing and for the ignition of “spontaneous” CSDs and precipitation of the migraine attack remain unknown.

Core microcircuits mediating feedback inhibition are essential for the dynamic maintaining of the cortical E/I balance, which is necessary for the transfer of information while preventing runaway excitation (and hence for the correct processing of sensory information). Besides the FS INs, these microcircuits also involve somatostatin-expressing (SOM) INs (Tremblay et al., 2016). Interestingly, SOM INs target the apical dendrites of PCs (where CSD initiates; Pietrobon and Moskowitz, 2014) and receive facilitating synaptic inputs from PCs (Reyes et al., 1998; Pala and Petersen, 2015; Tremblay et al., 2016; Stachniak et al., 2019). The large frequency-dependent facilitation of excitatory inputs onto SOM INs makes them sensitive to firing rate increases of even a small number of PCs, suggesting that the slow PC-SOM IN-PC feedback inhibition microcircuit prevents runaway excitation by applying an inhibitory tone that is proportional to the firing rate of PCs (rather than their synchrony level, as for the fast PC-FS IN-PC microcircuit; Tremblay et al., 2016). High-frequency firing of even a single PC can be sufficient to recruit SOM INs and give rise to frequency-dependent disynaptic inhibition (FDDI) on neighboring PCs (Kapfer et al., 2007; Silberberg and Markram, 2007). The SOM IN-mediated FDDI allows a firing rate-dependent recruitment of feedback inhibition even during sparse L2/3 activity. In sensory processing, SOM INs contribute to gain control and surround suppression (Adesnik et al., 2012) and, through regulation of dendritic spikes in apical dendrites, they play a critical role in gating top-down inputs and in context-dependent sensory perception (Murayama et al., 2009; Takahashi et al., 2016, 2020; Manita et al., 2017). Moreover, they are involved in synchronization of neuronal activity and in generation of cortical γ oscillations (Berger et al., 2010; Chen et al., 2017; Hilscher et al., 2017; Veit et al., 2017).

Given these important functions of SOM INs, here we investigated unitary excitatory and inhibitory synaptic transmission between L2/3 PCs and SOM INs and FDDI in FHM1 mice carrying the R192Q mutation, which in humans cause pure FHM, i.e., typical attacks of hemiplegic migraine without additional symptoms (Ophoff et al., 1996; Pietrobon, 2007).

## Materials and Methods

### Animals

Experiments were performed using homozygous knock-in mice carrying the R192Q FHM1 mutation (C57BL6J background; van den Maagdenberg et al., 2004) and expressing enhanced green fluorescent protein in a subset of SOM INs (FHM1-GIN mice). To obtain the FHM1-GIN mice, homozygous R192Q FHM1 mice were crossbred with homozygous FVB-Tg(GadGFP)45704Swn/J (GIN) mice (Oliva et al., 2000) and mice homozygous for both alleles were selected from the offspring of subsequent crossbreeds. Wild-type (WT)-GIN mice having the same genetic background as the FHM1-GIN mice were obtained by crossbreeding C57BL6J WT mice with homozygous GIN mice (Oliva et al., 2000).

In the following text, we will refer to WT-GIN and FHM1-GIN mice as WT and FHM1 mice, respectively. Animals were housed in specific pathogen-free conditions, maintained on a 12/12 h light/dark cycle, with free access to food and water. All experimental procedures involving animals and their care were performed in accordance with Italian national laws and policies (D.L. 26, March 14, 2014) and with the guidelines established by the European Community Council Directive (2010/63/UE), and were approved by the local authority veterinary services (AUT. MIN. 652/2015-PR).

### Slices

Acute thalamocortical slices were prepared from postnatal day (P)16–P21 mice of either sex as previously described (Tottene et al., 2009, 2019). Briefly, animals were deeply anesthetized with isoflurane and decapitated. The brain was quickly removed and dissected at the right angles to obtain thalamocortical slices, as described previously (Agmon and Connors, 1991); 300- to 350-mm-thick slices were then cut on the vibratome (Leica Microsystems, VT 1200S) in an ice-cold cutting solution (in mm as follows: 130 K gluconate, 15 KCl, 0.2 EGTA, 20 HEPES, 25 glucose, 2 kynurenic acid, and 0.05 minocycline, pH 7.4 with 5% CO_2_; Dugué et al., 2005). Slices were transferred for 1 min in a 95% O_2_/5% CO_2_ saturated solution containing (in mm as follows: 225 D-mannitol, 2.5 KCl, 1.25 NaH_2_PO_4_, 26 NaHCO_3_, 25 glucose, 0.8 CaCl_2_, 8 MgCl_2_, 2 kynurenic acid, and 0.05 minocycline) at room temperature, then in standard ACSF plus 50 nm minocycline at 30°C for at least 30 min, and finally at room temperature until being transferred to a submerged chamber where electrophysiological recordings were made at room temperature (21–24°C; within 6 h from cut).

### Electrophysiological recordings, experimental design, and data analysis

The recording chamber was mounted on the stage of an upright microscope (Eclipse E600FN, Nikon Instruments), and slices were continuously perfused with standard ACSF (in mm as follows: 125 NaCl, 2.5 KCl,1 MgCl_2_, 2 CaCl_2_, 25 NaHCO_3_, 1.25 NaH_2_PO_4_, and 25 glucose) saturated with 95% O_2_/5% CO_2_ at a flow rate of 3 ml/min using a peristaltic pump (Miniplus 3, Gilson).

Whole-cell patch-clamp recordings were made following standard techniques. Electrical signals were recorded through a Multiclamp 700B amplifier and digitized using an Axon Digidata 1550 interface and pClamp software (Molecular Devices). Pipette resistance was 3–5 mΩ. Currents were sampled at 10 kHz and filtered at 2 kHz. Access resistance was carefully monitored continuously throughout the experiments, and the recordings were discarded if the resistance changed ≥20% (or had access resistance >25 MΩ, without compensation). Data were not corrected for liquid junction potential.

The recordings were performed on L2/3 SOM INs and L2/3 PCs in the primary somatosensory barrel cortex (located at 45–65 µm in depth below the slice surface). Unitary synaptic transmission between SOM INs and PCs and between PCs was studied using double patch-clamp recordings in the presynaptic and postsynaptic neuron. Action potentials (APs) in the presynaptic neuron were induced in current clamp by injecting suprathreshold depolarizing current pulses (2–4 ms in duration). Unitary EPSPs (uEPSPs) were recorded in current clamp at −70 mV at PC-SOM IN synapses and at resting potential (−74 ± 1 mV, *n* = 14) at PC-PC synapses, while unitary IPSPs (uIPSCs) were recorded in voltage clamp (at 0 mV) at SOM IN-PC synapses. Short-term synaptic plasticity (STP) was studied using presynaptic AP trains at 20 Hz every 10 s at inhibitory SOM IN-PC synapses, at 25 Hz every 40 s and 100 Hz every 60 s at excitatory PC-SOM IN synapses and at 25 Hz every 30 s at PC-PC synapses. The kinetics of recovery from STP induced by 100-Hz AP trains were studied at PC-SOM IN synapses by injecting suprathreshold depolarizing currents to the presynaptic PC at different time intervals from the last AP in the 100-Hz train and recording the uEPSPs evoked in the postsynaptic SOM IN in 8–11 consecutive trials every 60 s for each time interval. The kinetics of decay of paired-pulse facilitation (PPF) at PC-SOM IN synapses were studied by injecting in the PC paired pulses of suprathreshold depolarizing current separated by different time intervals and recording the uEPSPs evoked in the SOM IN in 8–83 consecutive trials for each time interval. Connections in which rundown was larger than 15% during the first 5–7 min of recordings were excluded from the analysis. The FDDI was studied using dual patch-clamp recordings from nearby L2/3 PCs (soma distance ≤50 µm); trains of 10 APs at 100 Hz were elicited by suprathreshold current injections in one PC (PC1, in current clamp) and current was simultaneously recorded in the other PC (PC2) in voltage clamp at −40 mV in consecutive trials every 60 s.

For the study of the PC-SOM IN excitatory connection, the pipette internal solution for the postsynaptic SOM IN contained (in mm): 6 KCl, 129 KGluconate, 10 HEPES, 10 NaPhosphocreatine, 4 MgATP, 0,3 NaGTP, 0,2 EGTA, and 0.2% biocytin (pH 7.25 with KOH; LJP = −13 mV); the pipette for the presynaptic PC was filled with the same internal solution without EGTA. For the FDDI experiments and PC-PC connections, the KCl concentration was reduced to 2 mm (LJP = −13 mV). For the study of the SOM IN-PC inhibitory connection, KGluconate was substituted with CsMethanesulfonate (129 mm; pH 7.25 with CsOH) in the internal solution for the postsynaptic PC (LJP = −9 mV).

L2/3 PCs were identified according to their morphology under infrared differential interference contrast optics (using water-immersion objective 60×) and their spiking pattern in response to 500 ms pulses of depolarizing current of increasing intensity (Tottene et al., 2009). In a subset of experiments, the identification was confirmed by postfixation inspection of the biocytin-labeled neurons. SOM INs were identified on the basis of EGFP fluorescence, excited by Super High Pressure Mercury Lamp (Nikon C-SHG1 100W) and visualized with a CCD Camera (The Retiga ELECTRO) using a 60× water-immersion objective.

Postsynaptic events and disynaptic inhibitory currents were considered as events when the amplitude was at least 2.5-fold the baseline standard deviation. Amplitudes of the mean uIPSC in SOM IN-PC unitary connections and of the mean uEPSP in PC-SOM IN and PC-PC unitary connections were measured over a window of 1 ms around the peak of the mean uIPSC (obtained by averaging 31–103 sweeps at SOM IN-PC synapses) and of the mean uEPSP (obtained by averaging 30–74 and 16–40 sweeps at PC-SOM IN and PC-PC synapses, respectively). To obtain the responses evoked by each AP in the train after correction for the temporal summation, the decay phase of the mean uEPSP_j_ was fitted with a single or double exponential and the residual amplitude was subtracted from the peak of the mean uEPSP_j+1_. In the few PC-SOM IN connections in which the mean uEPSP_j_ was too small to be accurately fitted, the mean uEPSP was calculated by averaging the peak amplitudes of single trial uEPSPs, including failures; the uEPSPs were detected on the basis of the delay from the presynaptic AP and their time to peak (as obtained from the large uEPSPs measured at later stimuli in the train). In the case of the 100-Hz trains, the decay of the last mean uEPSP was fitted with a single or a double exponential and the temporal summation was corrected by using the time constants obtained from this fit.

The FDDI current was obtained by summing the outward currents elicited in PC2 by the high-frequency AP train in PC1 in the individual sweeps and by dividing by the total number of sweeps (which ranged from 15 to 59 in different experiments). The onset delay of FDDI was measured as the earliest time at which an outward current with IPSC kinetics was measured in PC2 after the onset of the AP train in PC1.

### Biocytin staining

After recording the slices were fixed in 150 mm phosphate buffer solution containing 3% paraformaldehyde for 2–5 d. After washing, quenching of endogenous peroxidase and permeabilization, the biocytin filled cells were marked by avidin and biotinylated horseradish peroxidase (Vectastain ABC elite, Vector Labs) and then colored by making them react with diaminobenzidine in the presence of hydrogen peroxide.

### Relationship between failures and average EPSP

According to a simple binomial model of synaptic transmission between two neurons, given *N* synaptic contacts with uniform release probabilities *p* and quantal amplitude *q*, the average EPSP (E) and the percentage of failures (F) are given by E = Npq and *F* = (1-p)^N^. For a synapse with low probability of release (*p* ≪ 1), F can be expanded in series: F ∼ 1-Np = 1-E/q. Thus, in a first approximation, for synapses with low probability of release, the relationship between the percentage of failures and the average EPSP is linear, and the coefficient is inversely proportional to the quantal amplitude. In order to minimize deviations from linearity, synapses with high release probability (*F* < 0.4) were excluded from the linear regression in Figure 2*C*.

### Statistics

Statistical analyses were performed with Statgraphics centurion XVII software (RRID: SCR_015248) and GraphPad Prism (RRID: SCR_002798). After assessing for normal distribution (using the Shapiro–Wilk normality test), comparison between two groups was made using two-tailed unpaired or paired *t* test for normally distributed data and the Mann–Whitney (MW) or Wilcoxon tests for nonparametric data. Equal variances were assumed. ANOVA for repeated measures was used for comparison of two groups of multiple measurements on the same cells. Pearson's χ^2^ test was used to test the difference between FDDI occurrence and PC-PC monosynaptic connections in the WT and FHM1. Correlation between mean uEPSP and percentage of failures was tested using Spearman's *r*. For the relationship between percentage of failures and mean uEPSP, the difference between the regression coefficients of the WT and FHM1 was tested with ANOVA, by considering a model with an interaction term (mean uEPSP × phenotype). Extra sum-of-squares *F* test was used to determine the best model to fit the PPF decay. The significance level was set to *p* < 0.05. Data are given in the text and figures as mean ± SEM.

The number *n* of observations (reported in the text and legends to figures) indicates the number of cells recorded from, and the number *N* indicates the number of mice from which the data were obtained (see figure legend for each experiment). No statistical methods were used to choose sample sizes that were estimated based on previous experience and are in line with those in the literature. No animals were excluded from the analysis.

## Results

**Figure 1.**
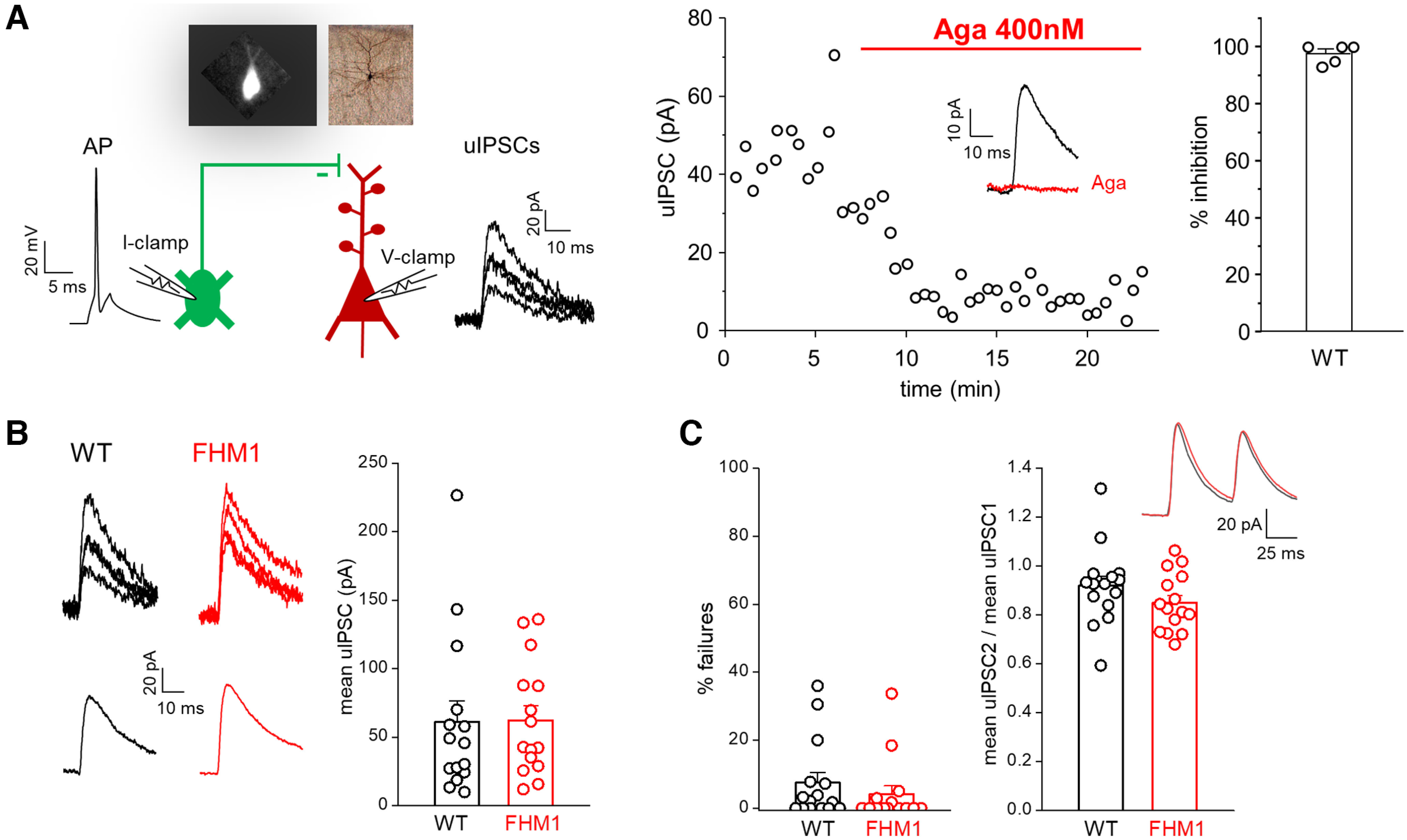
Inhibitory synaptic transmission between L2/3 SOM INs and L2/3 PCs is unaltered in FHM1 mice. ***A***, Left, Schematic diagram of the experimental configuration in which APs were elicited in a presynaptic L2/3 SOM IN in current clamp (I-clamp; −70 mV) and the uIPSCs evoked by the APs were recorded in a connected postsynaptic L2/3 PC in voltage clamp (V-clamp; 0 mV); representative traces of a presynaptic AP and postsynaptic uIPSCs are also shown. A representative epifluorescence image of an EGFP-positive SOM IN and a bright field image of a L2/3 PC after biocytin staining are shown on top of the diagram. Right, Time course of a representative experiment showing the uIPSCs evoked in a L2/3 PC before and after the application of a saturating concentration of the specific Ca_V_2.1 inhibitor ω-agatoxin-IVA (Aga; 400 nm) in WT mice. Inset, Mean uIPSC before (black) and after (red) bath application of Aga. Right, Percentages of inhibition of the mean uIPSC after application for 17 min of Aga (average value: 98 ± 2%; *n* = 5, *N* = 5). ***B***, Left, Representative uIPSCs (top) evoked in a L2/3 PC and corresponding mean uIPSC (bottom) in WT (black) and FHM1 (red) mice. Right, Amplitudes of the mean uIPSC in L2/3 PCs in WT (average value: 61 ± 15 pA, *n* = 15, *N* = 10) and FHM1 (average value: 62 ± 11 pA, *n* = 15, *N* = 13) mice. The average values of the mean uIPSC elicited in a L2/3 PC by APs in a connected presynaptic SOM IN are similar in FHM1 and WT mice (*f*_(28)_ = 0.44, *p* = 0.59 MW test). ***C***, Left, Percentages of failures in WT (average value: 7.5 ± 3.0%, *n* = 15, *N* = 10) and FHM1 (average value 4.1 ± 2.4% *n* = 15, *N* = 13) mice. Right, Paired pulse ratios (mean uIPSC2/mean uIPSC1; 50-ms interval) in WT (average value: 0.92 ± 0.04, *n* = 15, *N* = 10) and FHM1 (average value 0.85 ± 0.03, *n* = 15, *N* = 13) mice. Inset, Average mean uIPSCs elicited by a paired pulse in WT (black) and FHM1 (red) PCs. The percentage of failures and the paired pulse ratio are both similar in FHM1 and WT mice (*f*_(28)_ = 0.38, *p* = 0.16 MW test and *t*_(28)_ = 1.36, *p* = 0.19 *t* test, respectively).

We first investigated whether inhibitory neurotransmission at the synapses between L2/3 SOM INs and PCs is unaltered in FHM1 knock-in mice carrying the R192Q mutation (causing pure FHM in humans), as previously found at FS INs (and other non-SOM INs) synapses in FHM1 knock-in mice carrying either the R192Q or the S218L mutation (causing severe FHM with additional symptoms in humans; Tottene et al., 2009; Vecchia et al., 2014, 2015). We used dual patch-clamp recordings in acute slices of somatosensory cortex from WT and FHM1 mice expressing enhanced GFP in a subset of SOM INs (Oliva et al., 2000; Materials and Methods), most of which are L2/3 Martinotti cells that synapse onto the apical dendrites of L2/3 PCs (Ma et al., 2006; Fanselow et al., 2008). We studied unitary inhibitory synaptic transmission in connected pairs of L2/3 SOM INs and L2/3 PCs by measuring the uIPSCs evoked in the voltage-clamped (at 0 mV) postsynaptic PC by the APs generated in the presynaptic SOM IN by injection of depolarizing current pulses (Fig. 1*A*, left). The mean uIPSC was obtained by averaging the unitary responses (Fig. 1*B*). In a subset of paired recordings in WT slices, we measured the effect of saturating concentrations of the specific Ca_V_2.1 inhibitor ω-Agatoxin-IVA (400 nm) on the uIPSCs. The toxin almost completely suppressed the mean uIPSC (98 ± 2%, *n* = 5; Fig. 1*A*, right), thus showing that Ca_V_2.1 channels play a dominant role in initiating synaptic transmission at the inhibitory synapses between SOM INs and PCs in L2/3 of barrel cortex. An almost complete suppression of the mean uIPSC by ω-Agatoxin-IVA was obtained also in slices from FHM1 mice (96 ± 1%, *n* = 3).

Despite the dominant role of Ca_V_2.1 channels in controlling GABA release at the L2/3 SOM IN synapses, the probability of GABA release at these synapses was unaffected by the FHM1 mutation, as shown by the similar peak amplitudes of the mean uIPSC evoked in WT and FHM1 PCs (61 ± 15 pA in WT, *n* = 15 and 62 ± 11 pA in FHM1, *n* = 15, *f*_(28)_ = 0.44, *p* = 0.59 MW test; Fig. 1*B*) and the similar % of failures (i.e., the % of the presynaptic APs that failed to evoke a uIPSC in the PC: 7.5 ± 3.0% in WT, *n* = 15, and 4.1 ± 2.4% in FHM1, *n* = 15; *f*_(28)_ = 0.36, *p* = 0.16 MW test; Fig. 1*C*, left). The paired pulse ratio was also similar between the two genotypes (mean uIPSC2/mean uIPSC1 = 0.92 ± 0.04 in WT, *n* = 15 and 0.85 ± 0.03 in FHM1, *n* = 15; *t*_(28)_ = 1.36, *p* = 0.19 *t* test) consistently with an unaltered probability of GABA release at the L2/3 synapses between SOM INs and PCs in FHM1 mice (Fig. 1*C*, right).

The unaltered inhibitory synaptic transmission at SOM INs synapses in FHM1 mice (despite being initiated by Ca_V_2.1 channels) together with our previous findings of unaltered inhibitory transmission at FS INs and other non-SOM (likely 5HT3a receptor-expressing; Tremblay et al., 2016) INs synapses (Tottene et al., 2009; Vecchia et al., 2014, 2015) suggests that the lack of effect of FHM1 mutations on cortical inhibitory neurotransmission may be a general property regardless of the type of presynaptic IN.

**Figure 2.**
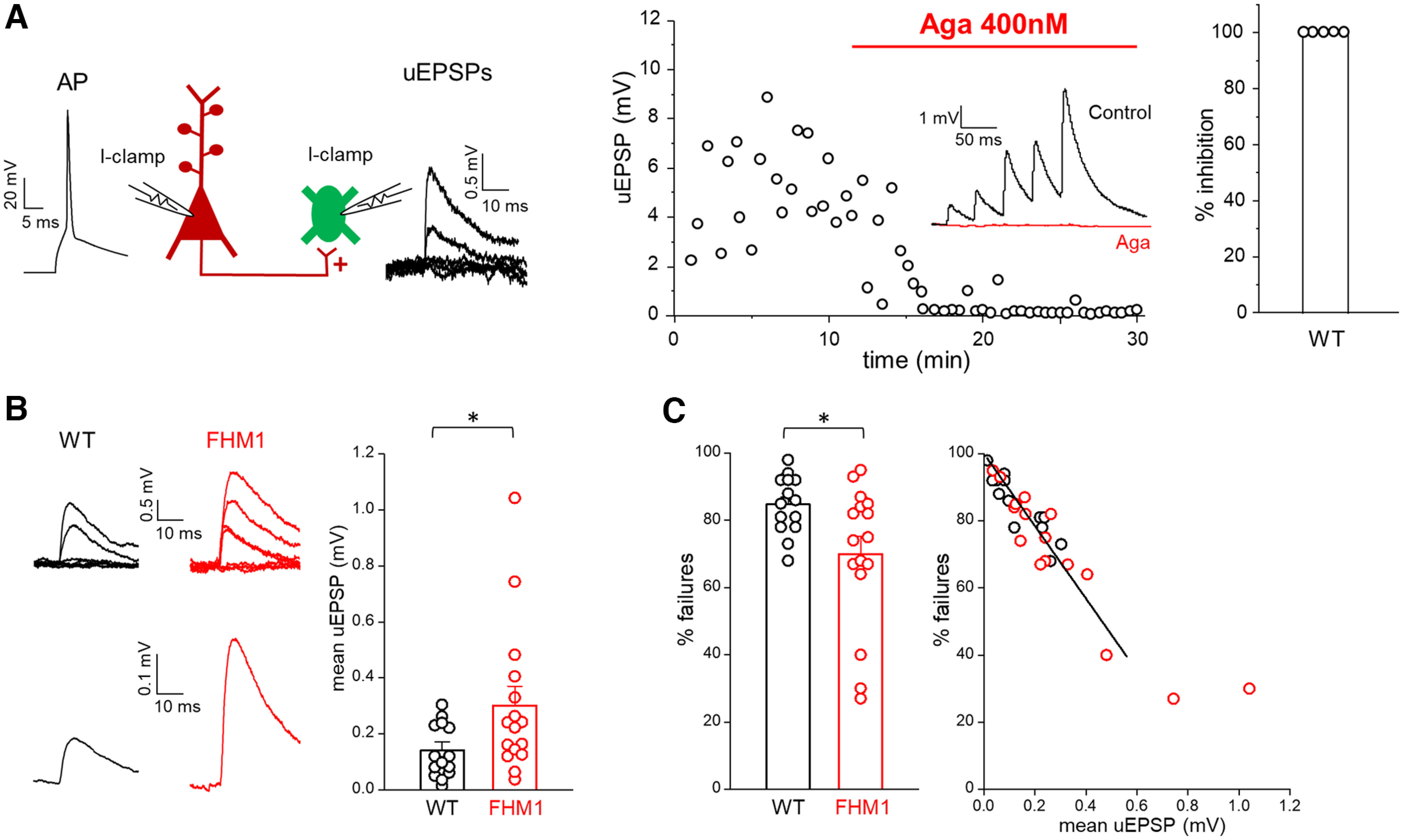
Excitatory synaptic transmission between L2/3 PCs and SOM INs is enhanced in FHM1 mice. ***A***, Left, Schematic diagram of the experimental configuration in which APs were elicited in a presynaptic L2/3 PC in current clamp (I-clamp; −70 mV) and the uEPSPs evoked by the APs were recorded in a connected postsynaptic L2/3 SOM IN in current clamp (I-clamp; −70 mV). Representative traces of a presynaptic AP and postsynaptic uEPSPs are also shown. Right, Time course of a representative experiment showing the uEPSPs evoked in a SOM IN before and after the application of a saturating concentration of Aga (400 nm) in a WT slice. Inset, Mean uEPSPs elicited in a SOM IN by 5 APs at 25 Hz in the presynaptic PC before (black) and after (red) bath application of Aga. Right, Percentages of inhibition of the mean uEPSP1 after application for 17 min of Aga (average value: 100 ± 0%, *n* = 5, *N* = 5). ***B***, Left, Representative uEPSPs (top) evoked in a SOM IN and corresponding mean uEPSP (bottom) in WT (black) and FHM1 (red) mice. Right, Amplitudes of the mean uEPSP in L2/3 SOM INs in WT (average value: 0.14 ± 0.03 mV, *n* = 14, *N* = 13) and FHM1 (average value: 0.30 ± 0.07 mV, *n* = 16, *N* = 14) mice. The average value of the mean uEPSP elicited in a SOM IN by stimulation of a connected presynaptic PC is 2.1 times larger in FHM1 compared with WT mice (*f*_(28)_ = 0.74, *p* = 0.025 MW test). ***C***, Left, Percentages of failures in WT (average value: 85 ± 2%, *n* = 14, *N* = 13) and FHM1 (average value 70 ± 5% *n* = 16, *N* = 14) mice. The percentage of failures is 18% lower in FHM1 compared with WT mice (*f*_(28)_ = 0.28, *p* = 0.039 MW test). Right, Percentages of failures measured in individual FHM1 and WT connections as a function of the mean uEPSPs measured in the same connections. Data (restricted to % failures ≥40%; see Materials and Methods) are best fitted by a shared linear function (for both the WT and FHM1) with slope −108 ± 5.

To study the effect of the FHM1 mutation on excitatory neurotransmission at the L2/3 PC-SOM INs synapses, we performed paired-patch clamp recordings in connected pairs of L2/3 PCs and SOM INs and measured in current-clamp (at −70 mV) the uEPSPs evoked in the postsynaptic SOM IN by application of suprathreshold stimuli to the presynaptic PC (Fig. 2*A*, left). The mean uEPSP was obtained by averaging the unitary responses (Fig. 2*B*). In a subset of paired recordings in WT slices, we measured the effect of saturating concentrations of ω-Agatoxin-IVA (400 nm) on the uEPSPs elicited in SOM INs on stimulation of PCs. The toxin completely suppressed the mean uEPSP (100 ± 0%, *n* = 5; Fig. 2*A*, right), thus showing that Ca_V_2.1 channels play a dominant role in initiating synaptic transmission at the excitatory synapses between PCs and SOM INs in L2/3 of barrel cortex. A nearly complete suppression of the mean uEPSP by ω-Agatoxin-IVA was also obtained in slices from FHM1 mice (99 ± 1%, *n* = 3).

The peak amplitude of the mean uEPSP was on average about two times larger in FHM1 compared with WT PC-SOM IN connections (0.30 ± 0.07 mV in FHM1, *n* = 16 vs 0.14 ± 0.03 mV in WT, *n* = 14, *f*_(28)_ = 0.74, *p* = 0.025 MW test; Fig. 2*B*). Consistent with an increased probability of AP-evoked glutamate release at the FHM1 synaptic contacts, the % failures was lower in FHM1 than in WT mice: 70 ± 5% in FHM1 (*n* = 16) versus 85 ± 2% in WT (*n* = 14; *f*_(28)_ = 0.28, *p* = 0.039 MW test; Fig. 2*C*, left). Consistent with an increased probability of release as underlying the enhanced mean uEPSP in FHM1 mice is also the similar inverse relationship between the % failures and the mean uEPSP in the individual FHM1 and WT connections (Spearman's *r* = −0.91, *p* < 0.0001, *n* = 30, 14 for WT and 16 for FHM1; Fig. 2*C*, right). For synapses with low probability of release, as for the PC-SOM IN synapse (Koester and Johnston, 2005), the % failures are approximately inversely proportional to the average EPSP (see Materials and Methods). We thus fitted the data with a linear model, considering both the mean uEPSP and the phenotype (WT or FHM1) as predictors for the percentage of failures. The slopes of the interpolating lines were not significantly different (mean uEPSP × phenotype, *F*_(1,25)_ = 0.001, *p* = 0.97, ANOVA), indicating a similar postsynaptic response to uniquantal release in FHM1 and WT mice.

The very small value of the mean uEPSP and very high value of % failures as well as the linear relationship between % failures and mean uEPSP in WT L2/3 PC-SOM IN synapses are consistent with and confirm the low probability of release that characterizes this synaptic connection, in contrast with the relatively high probability of release at the PC-FS IN connection (Reyes et al., 1998; Rozov et al., 2001; Koester and Johnston, 2005; Tottene et al., 2009; Pala and Petersen, 2015; Stachniak et al., 2019). The enhancement of the synaptic strength of excitatory synaptic transmission, because of an increased probability of glutamate release, appears then to be a general feature of cortical excitatory synapses in FHM1 knock-in mice regardless of their initial probability of release and postsynaptic target (Tottene et al., 2009, 2019).

**Figure 3.**
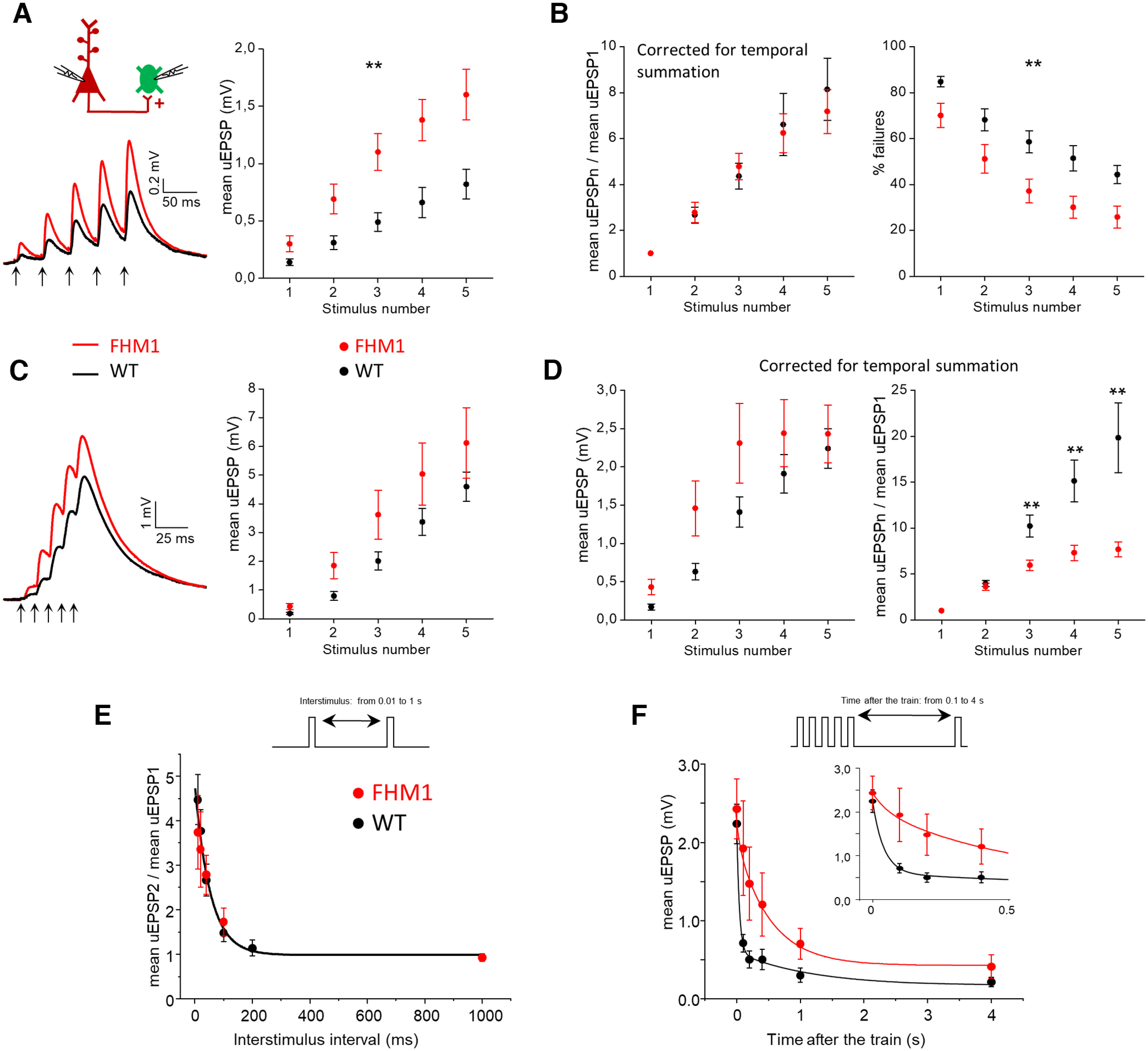
Frequency-dependent effect of the FHM1 mutation on short-term facilitation at the excitatory synapses between L2/3 PCs and SOM INs. ***A***, Left, Average mean uEPSP responses elicited in SOM INs by a train of 5 APs at 25 Hz in connected presynaptic PCs (indicated by arrows below the traces) in WT (black trace; *n* = 14, *N* = 11) and FHM1 (red trace, *n* = 16, *N* = 14) mice. Right, Peak amplitudes of the mean uEPSP elicited in SOM INs by each AP of the train (after correction for temporal summation) as a function of stimulus number in WT (black, *n* = 14) and FHM1 (red; *n* = 16; repeated-measures ANOVA *F*_(1,28)_ = 9.33, *p* = 0.005). ***B***, Left, Mean uEPSPs elicited in SOM INs by each AP of 25-Hz trains divided by the mean uEPSP elicited by the first AP in the train (mean uEPSPn/mean uEPSP1) as a function of stimulus number *n* in WT (black, *n* = 14) and FHM1 (red; *n* = 16) mice. Right, Percentage of failures in the SOM IN during 25-Hz AP trains as a function of stimulus number in WT (black, *n* = 14) and FHM1 (red; *n* = 16) mice (repeated-measures ANOVA *F*_(1,28)_ = 8.28, *p* = 0.008). At the PC-SOM IN synapse, the FHM1 mutation does not affect short-term facilitation of the mean uEPSPs elicited by five APs at 25 Hz and the gain-of-function of the mean uEPSPs produced by the mutation remains unaltered throughout the 25-Hz train. The percentage of failures remains lower in FHM1 mice throughout the 25-Hz AP train. ***C***, Left, Average mean uEPSP responses elicited in SOM INs by a train of 5 APs at 100 Hz in connected presynaptic PCs (indicated by arrows below the traces) in WT (black trace; *n* = 13, *N* = 10) and FHM1 (red trace, *n* = 16, *N* = 13) mice. Right, Peak amplitudes of the mean uEPSPs elicited in SOM INs as a function of stimulus number in WT (black, *n* = 13) and FHM1 (red; *n* = 16; repeated-measures ANOVA *F*_(1,27)_ = 2.02, *p* = 0.17). ***D***, Left, Peak amplitudes of the mean uEPSP elicited in SOM INs by each AP of 100-Hz trains in the presynaptic PC (after correction for temporal summation) as a function of stimulus number in WT (black, *n* = 13) and FHM1 (red; *n* = 16) mice (repeated-measures ANOVA *F*_(1,27)_ = 0.09, *p* = 0.21). Right, Mean uEPSPs elicited in SOM INs by each AP of 100-Hz trains (after correction for temporal summation) divided by the mean uEPSP elicited by the first AP in the train (mean uEPSPn/mean uEPSP1) as a function of stimulus number *n* in WT (black, *n* = 13) and FHM1 (red; *n* = 16) mice. For *n* = 3, 4, and 5, the mean uEPSPn/mean uEPSP1 is lower in FHM1 compared with WT mice (*t*_(27)_ = 3.44, *p* = 0.002, *t* test; *f*_(27)_ = 0.16, *p* = 0.002 MW; *t*_(27)_ = 3.47, *p* = 0.002, *t* test, respectively). Short-term facilitation of the mean uEPSPs elicited in SOM INs by five APs at 100 Hz in a presynaptic PC is reduced in FHM1 mice and the gain-of-function of the mean uEPSPs produced by the mutation decreases during the 100-Hz train. ***E***, Top, Schematic illustration of the stimulation protocol used to study the decay of the PPF of the mean uEPSPs elicited in SOM INs by two paired APs in the presynaptic PC, in which the time interval between the PC paired pulses is varied. Bottom, Paired pulse ratio of the mean uEPSPs elicited in SOM INs by paired pulse stimulation of the presynaptic PC (mean uEPSP2/mean uEPSP1) as a function of the time interval between the two pulses (interstimulus time interval) in WT (*n* = 8, *N* = 7) and FHM1 (*n* = 7, *N* = 6). The black line represents the shared parameter fit of the WT and FHM1 decay kinetics (time constant and amplitude with fit errors: τ = 54 ± 19 ms, amplitude = 4.8 ± 0.5). ***F***, Top, Schematic illustration of the stimulation protocol used to study the recovery from the facilitation of the mean uEPSPs elicited in SOM INs by five APs at 100 Hz in the presynaptic PC. In this protocol, suprathreshold stimuli were applied to the presynaptic PC at different time intervals from the last AP in the 100-Hz train. Bottom, Peak amplitude of the mean uEPSPs elicited in SOM INs by suprathreshold stimulations of a presynaptic PC at different time intervals from the last AP of the 100-Hz train in the same PC as a function of these time intervals in WT (black, *n* = 13, *N* = 10) and FHM1 (red, *n* = 16, *N* = 13) mice. The inset shows a magnification of the first 500 ms after the train. The continuous black line represents the best fit of the WT recovery kinetics, which required two exponentials (amplitude = 2.2 ± 0.1; time constant and % of the fast component with fit errors: τ_f_ = 41 ± 14 ms, A_f_ = 79 ± 16%; τ_s_ = 1182 ± 1833 ms for the slow component). The continuous red line represents the best fit with two exponentials with the time constant of the fast component fixed to the value of the time constant of decay of the PPF (amplitude = 2.4 ± 0.4; τ_f_ = 54 ms, A_f_ = 18 ± 13% and τ_s_ = 525 ± 428 ms).

To study the effect of the FHM1 mutation on short term plasticity (STP) at the L2/3 PC-SOM IN synapse, we measured the uEPSPs elicited in the postsynaptic SOM IN by trains of APs at 25 Hz and 100 Hz in the presynaptic PC (to approximate tonic and burst firing, respectively). In agreement with previous findings (Reyes et al., 1998; Rozov et al., 2001; Pala and Petersen, 2015; Stachniak et al., 2019), the WT synapse showed strong facilitation of the mean uEPSPs during 25-Hz trains (mean uEPSP2/mean uEPSP1 = 2.7 ± 0.4; mean uEPSP5/mean uEPSP1 = 8.1 ± 1.4, *n* = 14; Fig. 3*A*,*B*, black), and the % failures decreased during the train (from 85 ± 2% to 68 ± 5% at the second pulse to 44 ± 4% at the fifth pulse, *n* = 14; Fig. 3*B*, black), as expected for presynaptic mechanisms of short-term facilitation (STF) leading to increased probability of release with increasing stimulus number (Rozov et al., 2001; Stachniak et al., 2019). Also in agreement with previous findings (Fanselow et al., 2008; Pala and Petersen, 2015; Stachniak et al., 2019), the extent of facilitation was frequency-dependent, being much larger with 100 Hz (mean uEPSP2/mean uEPSP1 = 4.0 ± 0.3 and mean uEPSP5/mean uEPSP1 = 19.8 ± 3.8 after correction for temporal summation, *n* = 13; Fig. 3*D*, black) than 25-Hz trains.

In FHM1 mice, the mean uEPSPs elicited in SOM INs by each AP of 25-Hz trains were larger than in WT mice (repeated-measures ANOVA *F*_(1,27)_ = 9.33, *p* = 0.005; Fig. 3*A*) and the % failures remained smaller throughout the train (repeated-measures ANOVA *F*_(1,27)_ = 8.28, *p* = 0.008; Fig. 3*B*, right). Strikingly, both the PPF and the facilitation of the mean uEPSPs during the 25-Hz AP train were similar in FHM1 and WT mice (FHM1 mean uEPSP2/mean uEPSP1 = 2.8 ± 0.4, *n* = 16, *f*_(28)_ = 0.50, *p* = 0.98 MW; mean uEPSP5/mean uEPSP1 = 7.2 ± 1.0, *n* = 16, *f*_(28)_ = 0.42, *p* = 0.50 MW; Fig. 3*B*, left). In contrast, during AP trains at 100 Hz the extent of facilitation of the mean uEPSPs elicited by the third, fourth and fifth AP was smaller in FHM1 compared with WT mice (e.g., FHM1 mean uEPSP5/mean uEPSP1 = 7.7 ± 0.8 after correction for temporal summation, *n* = 16*; t*_(27)_ = 3.47, *p* = 0.002, *t* test; Fig. 3*D*, right), and the FHM1 mean uEPSP amplitudes elicited by each AP reached a plateau at the third AP (Fig. 3*D*, left). The PPF was similar in the two genotypes even at 100 Hz (FHM1 mean uEPSP2mean u/EPSP1 = 3.6 ± 0.4, *n* = 16, *f*_(27)_ = 0.38, *p* = 0.26 MW test). Without correction for the temporal summation of the mean uEPSPs (as occurs physiologically) the mean uEPSPs were larger in FHM1 compared with WT mice all along the 100-Hz train, as a trend, but the relative enhancement decreased from >100% at the first pulse to 33% at the fifth (Fig. 3*C*). Thus, in contrast with the unaltered gain-of-function of the mean uEPSPs produced by the FHM1 mutation during the 25-Hz stimulation, the gain-of-function decreases with increasing stimulus number during the 100-Hz stimulation.

There is evidence that the large STF at WT PC-SOM IN synapses depends on both the residual calcium and the activation of specific presynaptic calcium-permeable kainate receptors (Rozov et al., 2001; Stachniak et al., 2019). Both these STF mechanisms are expected to be enhanced in FHM1 mice because of the larger AP-evoked Ca^2+^ influx through mutant presynaptic Ca_V_2.1 channels and the larger probability of glutamate release at FHM1 synapses. On the other hand, one would also expect an enhancement of the short-term depression (STD) mechanisms of vesicle depletion and/or inactivation of the release sites after vesicle exocytosis, which are present at all synapses to an extent that correlates with the release probability (Dittman et al., 2000; Fioravante and Regehr, 2011). Our findings are consistent with a similar enhancement of the STF and STD mechanisms at FHM1 compared with WT synapses during 25-Hz AP trains, but a larger enhancement of STD compared with STF mechanisms during 100-Hz trains after the second stimulus.

To gain insights into the mechanisms underlying the frequency-dependent effect of the FHM1 mutation on STP, we studied the kinetics of decay of the PPF (mean uEPSP2/mean uEPSP1) and the kinetics of recovery from the facilitation of the mean uEPSPs after 100-Hz stimulation. To study the decay of PPF we measured the uEPSPs elicited in SOM INs by two presynaptic APs separated by different time intervals and evaluated the amount of facilitation of the mean uEPSP as a function of the interstimulus interval (Fig. 3*E*). As expected, the PPF decayed with the interstimulus time. We fitted the PPF decay with an exponential function and found that both the kinetics and the amplitude of PPF were not significantly different between WT and FHM1 mice, yielding a shared parameters fit as the best model to fit the data (Fig. 3*E*; τ = 54 ms, Amplitude = 4.76, *F*_(2,4)_ = 1.05, *p* = 0.429, *F* test, *n* = 4–8). The rate of decay of PPF at PC-SOM IN synapses is consistent with the rate of recovery of residual calcium measured at PC terminals (Koester and Sakmann, 2000; Jackman et al., 2016), suggesting a residual calcium-dependent PPF mechanism (Dittman et al., 2000; Jackman and Regehr, 2017) for both genotypes.

To study the recovery from the facilitation of the mean uEPSPs after 100-Hz stimulation we measured the uEPSPs elicited in the postsynaptic SOM IN by APs evoked in the presynaptic PC at different time intervals from the last AP in the 100-Hz train (Fig. 3*F*). The recovery from facilitation was slower in FHM1 mice; in these mice, 70% of the recovery occurred ∼1 s after the train in comparison with only 100 ms after the train in WT mice (Fig. 3*F*). Two exponentials were necessary to best fit the recovery kinetics of the WT mean uEPSP (*n* = 13; time constant and relative % amplitude: τ_f_ = 41 ms, A_f_ = 79% for the fast component; τ_s_ = 1182 ms for the slow component; Fig. 3*F*). The similar values of the time constant τ_f_ of the fast component of recovery from facilitation after a 100-Hz AP train and the time constant of decay of PPF suggest that the same residual calcium-dependent STP mechanism (Dittman et al., 2000; Jackman and Regehr, 2017) likely underlies the PPF and most of the facilitation after short high-frequency stimulation at WT PC-SOM synapses. However, an additional longer lasting STP mechanism specifically associated with the 100-Hz AP train accounts for a relatively small component of the recovery from facilitation after high-frequency stimulation in WT mice. In FHM1 mice, this slow component is much larger, as shown by the parameters of the biexponential fit of the kinetics of recovery after the 100-Hz train (A_s_ = 82% and τ_s_ = 525 ms; Fig. 3*F*) in which the time constant of the fast exponential component was constrained to the value of the shared time constant of decay of the PPF in the two genotypes (τ = 54 ms; Fig. 3*E*). This suggests that at FHM1 PC-SOM IN synapses the recovery from facilitation appears dominated by the longer lasting STP mechanism specifically associated with the 100-Hz AP train. One would expect an enhancement of this mechanism at FHM1 synapses if it was calcium-dependent with a rate of recovery slower than that of the PPF mechanism. If one assumes that the slower component of the recovery from facilitation after a 100-Hz train reflects at least in part the rate of recovery from STD mechanisms (Fioravante and Regehr, 2011), a larger enhancement of STD compared with STF at FHM1 synapses might also contribute to the slower recovery in FHM1 mice. To summarize, we found that the response of SOM INs to high-frequency activation of PCs was enhanced during the AP bursts and remained potentiated for longer time intervals after the bursts in FHM1 compared with WT mice.

**Figure 4.**
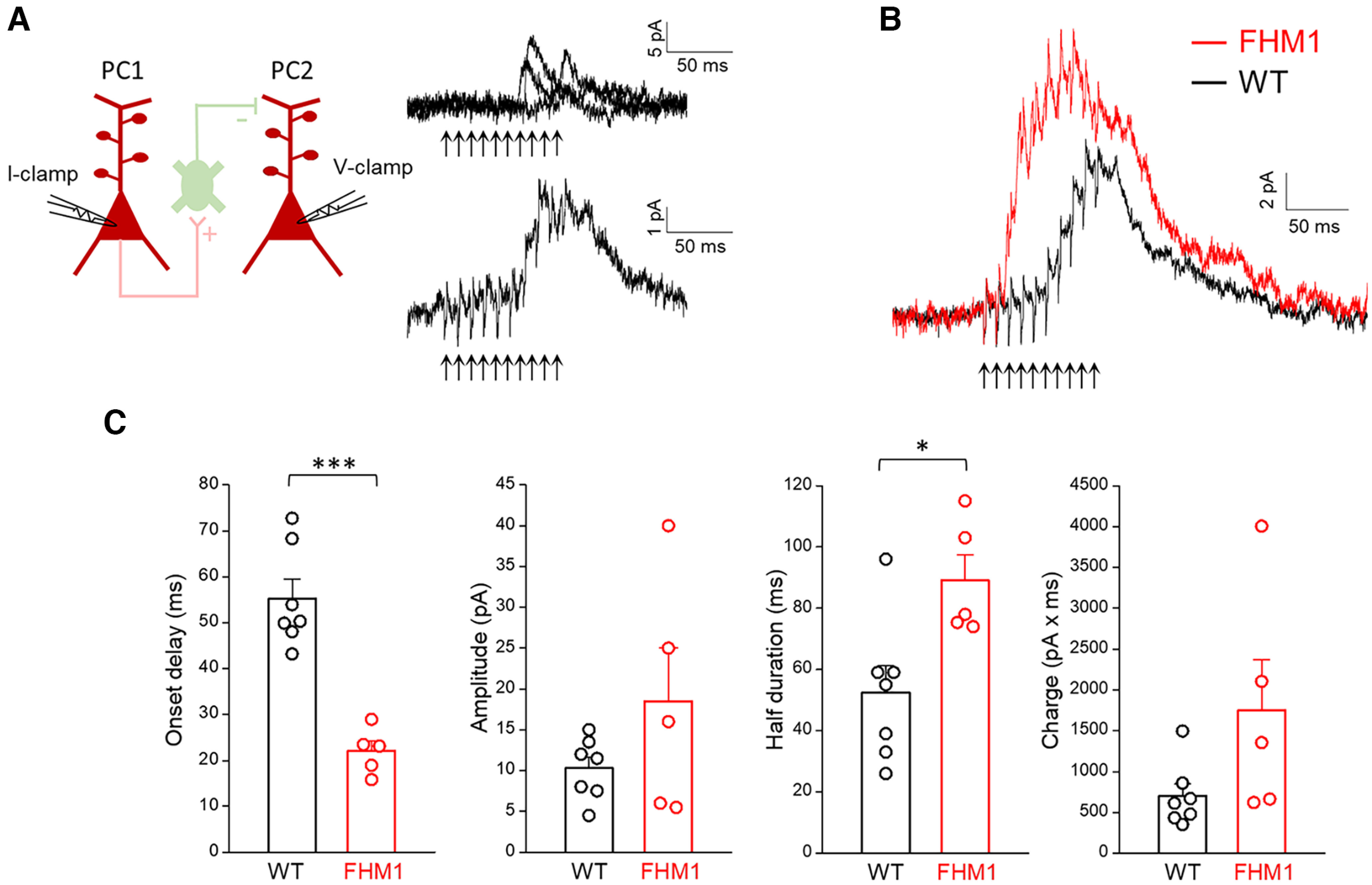
FDDI has an earlier onset and is enhanced in FHM1 mice. ***A***, Left, Schematic diagram of the experimental configuration in which 100-Hz trains of APs were elicited in a L2/3 PC (PC1) in current clamp (I-clamp) and current was simultaneously recorded in a nearby L2/3 PC (PC2) in voltage clamp (V-clamp; −40 mV). Right, Traces from a representative WT experiment in which ten APs in PC1 (indicated by the arrows below the traces) evoked outward inhibitory currents in the simultaneously recorded PC2: three superimposed selected sweeps are shown on top and the average of 22 sweeps is shown below. The latter represents the FDDI, i.e., the disynaptic inhibitory current produced by the recruitment of connected SOM INs (postsynaptic to PC1 and presynaptic to PC2, as indicated in the top diagram) by the high-frequency train in PC1 (Kapfer et al., 2007). ***B***, Average disynaptic inhibitory currents recorded in PC2 in WT (black trace; *n* = 7, *N* = 6) and FHM1 (red trace; *n* = 5, *N* = 5) mice. The arrows below the traces indicate the time of the APs in PC1. ***C***, Onset delay, amplitude, half duration and charge of the FDDI in WT (*n* = 7, *N* = 6) and FHM1 (*n* = 5, *N* = 5) mice. The onset delay of FDDI is 60% shorter in FHM1 (22 ± 2 ms) compared with WT (55 ± 4 ms; *t*_(10)_ = 6.21, *p* = 0.0001 *t* test) mice. The amplitude of FDDI is 85% larger in FHM1 (18.5 ± 6.5 pA) compared with WT (10 ± 1 pA) mice, but the difference is not statistically significant (*t*_(10)_ = 1.46, *p* = 0.17, *t* test). The half duration of FDDI is 71% larger in FHM1 (89 ± 8 ms) compared with WT (52 ± 9 ms; *t*_(10)_ = 2.90, *p* = 0.016, *t* test) mice. The FDDI charge is 2.5 times larger in FHM1 (1748 ± 625 pA/ms) compared with WT (699 ± 147 pA/ms; *t*_(10)_ = 1.92, *p* = 0.08, *t* test) mice.

Because of the large frequency-dependent facilitation of excitatory inputs onto SOM INs and the specific intrinsic properties of these INs (high input resistance and slow time constant favoring EPSP summation), high-frequency firing of even a single PC can be sufficient to recruit SOM INs and give rise to delayed disynaptic inhibition on neighboring PCs (the so called FDDI; Kapfer et al., 2007; Silberberg and Markram, 2007). To investigate whether and how the FDDI is altered in FHM1 mice, we performed dual patch-clamp recordings from nearby L2/3 PCs (soma distance ≤ 50 µm) in somatosensory cortex of WT and FHM1 mice. We triggered 10 APs at 100 Hz in a PC (PC1) and simultaneously recorded the current in a “target” PC (PC2; voltage clamped at −40 mV). In WT slices, high-frequency trains in PC1 evoked outward inhibitory currents in PC2 in a fraction of experiments (7.1%: 9 out of 126; Fig. 4*A*). Consistent with the properties of the L2/3 FDDI mediated by SOM INs described previously (Kapfer et al., 2007), the mean disynaptic inhibitory current in WT mice developed slowly after a delay of 55 ± 4 ms (*n* = 7) from the onset of the AP train in PC1, reached its peak value at the last AP (peak amplitude 10 ± 1 pA, *n* = 7) and then slowly decayed (duration at half amplitude: 52 ± 9 ms; Fig. 4*B*,*C*, black).

In FHM1 mice, the onset latency with which the disynaptic inhibitory current developed in PC2 after the first AP in PC1 was reduced by 60% compared with WT mice (22 ± 2 ms, *n* = 5, *t*_(10)_ = 6.21, *p* = 0.0001 *t* test; Fig. 4*B*,*C*). Moreover, the disynaptic inhibition lasted 71% longer (duration at half amplitude: 89 ± 8 ms, *n* = 5, *t*_(10)_ = 2.90, *p* = 0.016 *t* test; Fig. 4*B*,*C*). The peak amplitude of the disynaptic inhibitory current was reached earlier along the train (at the seventh AP, after which it tended to decline) and was (85%) larger in FHM1 compared with WT mice (18.5 ± 6.5 pA, *n* = 5, *t*_(10)_ = 1.46, *p* = 0.17 *t* test), but the difference did not reach statistical significance, because of the large variability (Fig. 4*B*,*C*). As a consequence of both its larger duration and larger amplitude, the integral of the disynaptic inhibitory current (i.e., the disynaptic inhibitory charge) was 2.5 times larger in FHM1 compared with WT mice (FHM1: 1748 ± 625 pA/ms vs WT: 699 ± 147 pA/ms, *t*_(10)_ = 1.92, *p* = 0.08 *t* test; Fig. 4*C*). FDDI was observed in a similar fraction of experiments in FHM1 (6.4%: 11 out of 172) and WT mice (Pearson's χ^2^, effect size = 0.99, *p* = 0.81). The earlier onset, longer duration, and tendency to larger amplitude of FDDI in FHM1 mice indicate that, as a consequence of the gain-of-function of excitatory transmission at the L2/3 PC-SOM synapses, a shorter train of high-frequency APs is necessary and sufficient to recruit SOM INs in FHM1 compared with WT mice and that the recruitment of SOM INs remains larger than in WT during most of the high-frequency train. Thus, our data support the conclusion that the slow disynaptic feedback inhibition mediated by SOM INs in L2/3 of the barrel cortex is enhanced in FHM1 mice.

**Figure 5.**
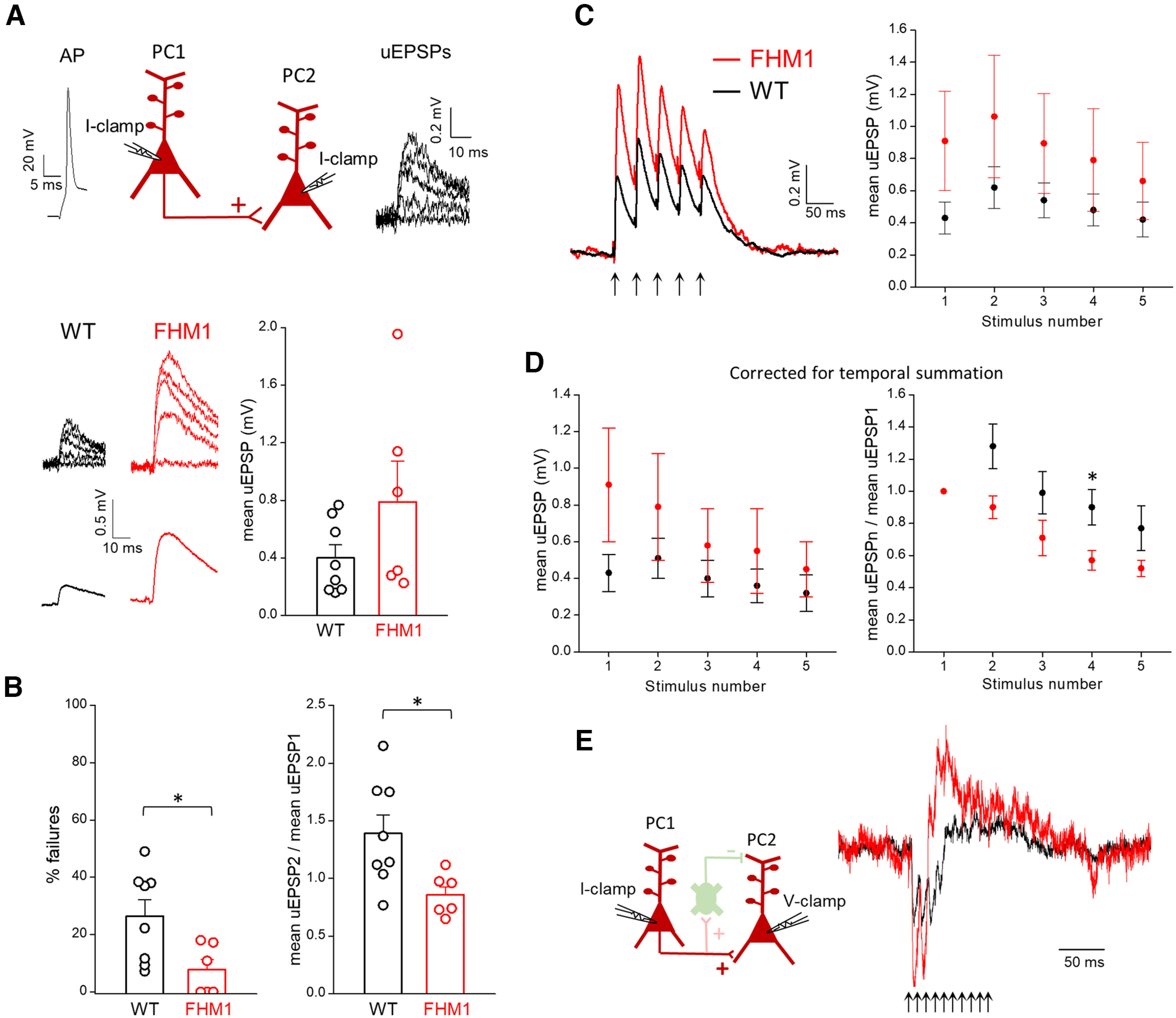
Excitatory synaptic transmission between L2/3 PCs is enhanced in FHM1 mice. ***A***, Top, Schematic diagram of the experimental configuration in which APs were elicited in a presynaptic L2/3 PC in current clamp (I-clamp) and the uEPSPs evoked by the APs were recorded in a connected postsynaptic L2/3 PC in current clamp (I-clamp). Representative traces of a presynaptic AP and postsynaptic uEPSPs are also shown. Bottom, Left, Representative uEPSPs (upper traces) evoked in the postsynaptic PC and corresponding mean uEPSPs (lower traces) in WT and FHM1 mice. Right, Amplitudes of the mean uEPSPs in WT (average value: 0.40 ± 0.09 mV, *n* = 8, *N* = 8) and FHM1 (average value: 0.79 ± 0.28 mV, *n* = 6, *N* = 5) mice. The mean uEPSP elicited in PCs by stimulation of a connected presynaptic PC is approximately two times larger in FHM1 compared with WT mice, but the difference is not statistically significant (*t*_(12)_ = 1.54, *p* = 0.15, *t* test). ***B***, Left, Percentages of failures in WT (average value: 27 ± 6%, *n* = 8, *N* = 8) and FHM1 (average value 8 ± 4% *n* = 6, *N* = 5) mice. Right, Paired pulse ratios (mean uEPSP2/mean uEPSP1, after correction for temporal summation; 40-ms interval) in WT (average value: 1.4 ± 0.2, *n* = 8, *N* = 8) and FHM1 (average value 0.86 ± 0.07, *n* = 6, *N* = 5) mice. The percentage of failures and the paired pulse ratio are both smaller in FHM1 compared with WT mice (by 70%, *f*_(12)_ = 0.83, *p* = 0.04 MW test and 63%, *t*_(12)_ = 2.61, *p* = 0.02 *t* test, respectively). ***C***, Left, Average mean uEPSP responses elicited in postsynaptic PCs by a train of 5 APs at 25 Hz in connected presynaptic PCs (indicated by arrows below the traces) in WT (black trace; *n* = 7, *N* = 7) and FHM1 (red trace, *n* = 5, *N* = 5) mice. Right, Peak amplitudes of the mean uEPSP elicited in postsynaptic PCs as a function of stimulus number in WT (black, *n* = 7) and FHM1 (red; *n* = 5; repeated-measures ANOVA *F*_(1,10)_ = 1.60, *p* = 0.23). ***D***, Left, Peak amplitudes of the mean uEPSP elicited in postsynaptic PCs by each AP of 25-Hz trains in the presynaptic PC (after correction for temporal summation) as a function of stimulus number in WT (black, *n* = 7, *N* = 7) and FHM1 (red; *n* = 5, *N* = 5) mice (repeated-measures ANOVA *F*_(1,10)_ = 1.26, *p* = 0.29). Right, Mean uEPSPs elicited in PCs by each AP of 25-Hz trains (after correction for temporal summation) divided by the mean uEPSP elicited by the first AP in the train (mean uEPSPn/mean uEPSP1) as a function of stimulus number *n* in WT (black, *n* = 7) and FHM1 (red; *n* = 5) mice. STD of the mean uEPSPs during the 25-Hz train is larger in FHM1 compared with WT mice and the gain-of-function of the mean uEPSPs produced by the mutation decreases during the train. ***E***, Same experimental configuration as in Figure 4*A*, but PC1 and PC2 are connected and therefore, a 100-Hz train of 10 APs in PC1 produces both direct monosynaptic excitation and delayed disynaptic inhibition of PC2, as shown by the representative traces of the average current recorded in a WT PC2 cell (black trace) and a FHM1 PC2 cell (red trace). The traces were normalized to make the EPSC elicited by the first AP in the FHM1 trace to be two times larger than that in the WT trace (compare panel ***A***).

In ∼10% of the dual patch-clamp recordings from nearby L2/3 PCs (13 out of 126 in WT slices), each AP in the PC1 train elicited an inward excitatory current in PC2 with a short delay consistent with a monosynaptic connection between the two PCs. Monosynaptic excitation was observed in a similar fraction of experiments in slices from FHM1 mice (4.8%: 9 out of 186, not significantly different from in WT, Pearson's χ^2^, effect size = 0.95, *p* = 0.09). In these experiments, the mean uEPSP (obtained by averaging the unitary responses evoked in the postsynaptic PC by an AP elicited in the presynaptic PC) was, on average, about two times larger in FHM1 compared with WT mice (FHM1: 0.79 ± 0.28 mV, *n* = 6 vs WT: 0.40 ± 0.09 mV, *n* = 8, *t*_(12)_ = 1.54, *p* = 0.15 *t* test; Fig. 5*A*). Although, because of the large variability, the mean uEPSP difference did not reach statistical significance, the probability of glutamate release at L2/3 PC-PC synapses was significantly increased in FHM1 mice, as shown by the significantly lower % of failures in FHM1 compared with WT mice (FHM1: 8 ± 4%, *n* = 6 vs WT: 27 ± 6%, *f*_(12)_ = 0.83, *p* = 0.04, MW test; Fig. 5*B*, left). Consistent with this, the paired pulse ratio was significantly lower in the mutant mice (FHM1: mean uEPSP2/mean EPSP1 = 0.86 ± 0.07, *n* = 6 vs WT: mean uEPSP2/mean uEPSP1 = 1.4 ± 0.2 after correction for temporal summation, *n* = 8, *t*_(12)_ = 2.61, *p* = 0.02, *t* test), revealing paired-pulse depression in FHM1 PC-PC synapses in contrast with PPF in WT synapses (Fig. 5*B*, right). In response to the subsequent pulses in trains of APs at 25 Hz in the presynaptic PC, also the WT synapses showed STD (after correction for temporal summation; Fig. 5*D*), indicating the coexistence at PC-PC synapses of mechanisms of STD, such as vesicle depletion and/or inactivation of the release sites after vesicle exocytosis, and mechanisms of STF such as those dependent on residual calcium (as occurs at many synapses; Dittman et al., 2000; Fioravante and Regehr, 2011; Jackman and Regehr, 2017). As expected for an increased probability of glutamate release, STD was larger at FHM1 compared with WT PC-PC synapses (Fig. 5*D*, right); as a consequence, the mean uEPSPs elicited by the last AP of the 25-Hz train were similar in the two genotypes (Fig. 5*D*, left). Without correcting the temporal summation (as occurs physiologically), the mean uEPSPs were larger in FHM1 compared with WT mice all along the 25-Hz train, as a trend, but the relative enhancement decreased from >100% at the first pulse to 57% at the fifth pulse (Fig. 5*C*).

Four paired recordings from nearby L2/3 PCs, in which we recorded the current elicited in PC2 by 10 APs at 100 Hz in PC1, revealed both monosynaptic excitation and FDDI in PC2 (Fig. 5*E*). The representative traces in Figure 5*E* show that the FDDI effectively curtails the excitatory synaptic currents elicited by the high-frequency train and, hence, narrows the time window of integration of the excitatory inputs in both WT and FHM1 mice. The reduced onset latency of FDDI in FHM1 compared with WT mice results in a shorter duration of the excitation of the postsynaptic PC, and hence in a shorter time window of integration of the enhanced excitatory inputs elicited by high-frequency bursts. Together with the enhanced and prolonged FDDI, which more effectively dampens excitatory inputs outside the integration time window, this would increase the temporal precision of spiking in PCs.

## Discussion

A first novel finding of this study is that inhibitory synaptic transmission at cortical L2/3 SOM INs synapses is unaltered in FHM1 mice, despite being initiated by Ca_V_2.1 channels. This, together with our previous finding of unaltered inhibitory neurotransmission at cortical FS and non-SOM (likely 5HT3aR-expressing) INs synapses (Tottene et al., 2009; Vecchia et al., 2014, 2015), supports the general conclusion that FHM1 mutations do not directly affect cortical inhibitory neurotransmission. In light of the findings of Vecchia et al. (2014), including the finding of unaltered Ca_V_2.1 current in cortical multipolar (FS and non-SOM) INs from FHM1 knock-in mice in contrast with the enhanced current density and left-shifted activation of the mutant Ca_V_2.1 channels in cortical PCs (Tottene et al., 2009), a likely mechanism underlying the unaltered cortical inhibitory neurotransmission in FHM1 mice is the expression in inhibitory INs of specific Ca_V_2.1 channels whose gating is barely affected by the FHM1 mutation. While the molecular mechanisms underlying the differential modulation of the Ca_V_2.1 channels expressed in excitatory and inhibitory cortical neurons remain unknown, possible mechanisms include the expression of different splicing variants (Adams et al., 2009) and/or the expression of different auxiliary subunits (Müllner et al., 2004). An important general implication is that neuron subtype-specific and synapse-specific effects (cf. also Fioretti et al., 2011) may help to explain why a mutant calcium channel that is widely expressed in the nervous system produces the specific brain dysfunctions underlying FHM (Pietrobon, 2013).

Another novel finding is that excitatory synaptic transmission at cortical L2/3 PC synapses onto both SOM INs and PCs is enhanced in FHM1 mice and this is accompanied by alterations in STP at PC-PC but not at PC-SOM IN synapses during trains of APs at 25 Hz. The enhancement of synaptic strength is because of an increased probability of glutamate release at both PC-PC and PC-SOM IN synapses. This, together with previous findings of enhanced excitatory neurotransmission at several FHM1 brain synapses (Tottene et al., 2009, 2019; Adams et al., 2010; Di Guilmi et al., 2014; Dilekoz et al., 2015; Eikermann-Haerter et al., 2015; Vecchia et al., 2015) supports the conclusion that an enhanced excitatory synaptic transmission, because of increased probability of glutamate release, is a general property in FHM1, regardless of the identity of the presynaptic neuron and postsynaptic target. The differential effect of the FHM1 mutation on STP at PC-PC and PC-SOM IN synapses, together with the differential effect on STP at thalamocortical synapses on principal neurons and FS INs (Tottene et al., 2019), supports the conclusion that the effect of the FHM1 mutation on STP may differ at different excitatory synapses depending on their specific plasticity mechanisms, e.g., on whether and to what extent these mechanisms depend on initial release probability (such as vesicle depletion-dependent STD) or on AP-evoked calcium influx (such as residual calcium-dependent STF; Dittman et al., 2000; Fioravante and Regehr, 2011).

Because of the unaltered STF at PC-SOM IN synapses and increased STD at PC-PC synapses in FHM1 mice, the gain-of-function of excitatory neurotransmission remains constant during short 25-Hz trains of APs at PC-SOM synapses but decreases with increasing pulse number at PC-PC synapses. Considering the high degree of reciprocal interconnectivity between PCs and SOM INs and the much higher connectivity between SOM INs and PCs than among PCs in L2/3 (Lefort et al., 2009; Fino and Yuste, 2011; Jouhanneau et al., 2015; Urban-Ciecko and Barth, 2016) and that SOM INs are responsible for most of the feedback inhibition elicited in L2/3 PCs by synchronous firing of relatively few PCs (Hakim et al., 2018), one predicts that during repetitive PC activity at 25 Hz, the increase in disynaptic feedback inhibition produced by the FHM1 mutation may become larger than the increase in monosynaptic excitation. Hence, the cellular E/I balance in L2/3 PCs may become skewed toward inhibition in FHM1 mice, as we have shown to occur in L4 principal neurons during repetitive thalamic activity (Tottene et al., 2019).

A last novel finding of this study is that the SOM IN-mediated FDDI (i.e., the disynaptic inhibition produced on neighboring L2/3 PCs as a consequence of recruitment of SOM INs by a high-frequency train of APs in even a single L2/3 PC; Kapfer et al., 2007), is enhanced, lasts longer and requires a lower number of APs to be initiated in FHM1 compared with WT mice. This reflects the gain-of-function of excitatory transmission at the L2/3 PC-SOM IN synapses, whereby the temporal summation of a lower number of the facilitating EPSPs is sufficient to recruit SOM INs and the recruitment of SOM INs is larger in FHM1 mice. Thus, in contrast with the relatively long high-frequency bursts necessary to induce FDDI in WT slices, even short high-frequency bursts, similar to those observed physiologically in awake mice (de Kock and Sakmann, 2008; Yu et al., 2019), can induce FDDI in FHM1 slices. Moreover, considering that FDDI increases supralinearly with increasing number of active L2/3 PCs and can be triggered in WT mice by brief synchronous high-frequency bursts in only few PCs (Kapfer et al., 2007; Berger et al., 2010), shorter (or lower frequency) synchronous bursts in fewer active PCs are expected to trigger FDDI in FHM1 compared with WT mice.

Our data show that the slow disynaptic feedback inhibition mediated by SOM INs in L2/3 of the barrel cortex is enhanced in FHM1 mice. Of course, the finding of enhanced strength of synaptic transmission at L2/3 PC-PC synapses predicts increased recurrent excitation in FHM1 mice. In principle, this may lead to hyperexcitability or even runaway excitation if the enhancement of intracortical recurrent (and thalamocortical; Tottene et al., 2019) synaptic excitation overcome the enhancement of feedback (and feedforward) inhibition. However, this does not occur in FHM1 cortical slices, as indicated by the reduced cortical recurrent network activity induced by repetitive thalamic firing in FHM1 compared with WT mice (Tottene et al., 2019) and the subtle changes in the spontaneous upstate firing rates of L2/3 PCs (Fabbro and Pietrobon, unpublished results). Apparently, the increase in slow disynaptic feedback inhibition mediated by SOM INs (likely together with the increase in fast disynaptic feedback and feedforward inhibition mediated by FS INs (Tottene et al., 2009, 2019) may efficiently counteract the increased recurrent excitation, and may even prevail in certain conditions.

The feedback inhibition mediated by the PC-SOM IN-PC microcircuit has a critical role in the generation of long-range γ oscillations in response to optogenetic activation of L2/3 PCs in cortical slices (Hakim et al., 2018) and in the generation of size-dependent γ oscillations in the primary visual cortex, V1, of awake mice (Chen et al., 2017; Veit et al., 2017). The V1 PC-SOM IN-PC microcircuit also plays an important role in surround suppression (Adesnik et al., 2012). Since an increased recruitment of SOM INs is fundamental for both γ band synchronization and surround suppression (Adesnik et al., 2012; Veit et al., 2017; Hakim et al., 2018) our findings suggest that these phenomena might be enhanced in FHM1. Interestingly, there is evidence for increased perceptual center-surround suppression for drifting visual stimuli (Battista et al., 2011) and increased γ band activity in visual-evoked responses (Coppola et al., 2007) in interictal migraineurs, as well as for correlation between γ oscillations in V1 and visual discomfort (Keil et al., 2007) and γ oscillations in S1 and subjective pain perception in humans (Gross et al., 2007). A causal link between specific enhancement of γ oscillations in S1 and facilitation of nociceptive sensitivity was recently shown in mice (Tan et al., 2019).

*In vivo*, the recruitment of SOM INs can remarkably change depending on behavioral states and context, because SOM INs are very sensitive to neuromodulation and are the main target of vasoactive intestinal peptide-expressing (VIP) INs, which are mainly activated by corticocortical feedback projections from higher order cortices and/or neuromodulation (Lee et al., 2013; Pfeffer et al., 2013; Pi et al., 2013; Tremblay et al., 2016; Urban-Ciecko and Barth, 2016). Being activated by states of increased arousal such as whisking, movement, attention and punishment/reward, the VIP IN-SOM IN-PC disinhibitory microcircuit is an important mechanism of top-down modulation of sensory processing and gain control (Lee et al., 2013; Pi et al., 2013; Fu et al., 2014; Zhang et al., 2014; Williams and Holtmaat, 2019; Yu et al., 2019). Although the effect of FHM1 mutations on the activation of this disinhibitory microcircuit remains to be studied, the generally enhanced neurotransmission at FHM1 excitatory synapses makes one predict an increased recruitment of VIP INs and a consequent larger inhibition of SOM INs by VIP INs in FHM1 compared with WT mice. Thus, since SOM INs participate in interacting and competing microcircuits whose alterations in FHM1 may have opposite effects on PC firing (cf. also the inhibitory effect of SOM INs on other INs Pfeffer et al., 2013), it is quite difficult to predict how the FHM1 mutations alter the complex dynamics of the cortical circuits *in vivo* during sensory processing. It is also difficult to predict how the mutations affect the generation of NMDA and Ca spikes in the apical dendrites of PCs in sensory cortices. These long-lasting dendritic spikes have a key role in amplification of distal L1 top-down synaptic inputs, and are particularly interesting in the context of migraine, given their important role in sensory gain and context-dependent sensory perception (Major et al., 2013; Smith et al., 2013; Manita et al., 2015, 2017; Takahashi et al., 2016, 2020) and given that the NMDA receptors critically involved in CSD initiation are located on the apical dendrites of PCs (Pietrobon and Moskowitz, 2014). Interestingly, FHM2 knock-in mice, having reduced rate of glutamate clearance at cortical excitatory synapses (Leo et al., 2011; Capuani et al., 2016; Parker et al., 2021), show facilitation of NMDA spikes in L5 PC tuft dendrites in cingulate cortex slices, which is correlated with enhanced sensitivity to a migraine-relevant head pain trigger (Romanos et al., 2020); they also show enhanced activation of extrasynaptic GluN1-N2B NMDA receptors in L2/3 PC apical dendrites in barrel cortex slices, whose inhibition rescues the facilitation of experimental CSD (Crivellaro et al., 2021). *In vivo*, opposite effects of FHM1 mutations on the generation of NMDA and Ca spikes are predicted on the basis of the enhanced FDDI in FHM1 mice (Murayama et al., 2009) and the predicted enhanced excitatory transmission at the L1 top-down synaptic inputs and enhanced activation of the disinhibitory VIP IN-SOM IN-PC microcircuit. The prevailing outcome will strongly depend on behavioral state and context.

As a whole, our results support the general conclusion that FHM1 mutations enhance excitatory synaptic transmission without directly affecting inhibitory synaptic transmission regardless of the identity of the presynaptic and postsynaptic neuron, although the latter may determine how the mutations affect STP. Via increased recruitment of SOM INs, the disynaptic feedback inhibition mediated by these INs is enhanced and is triggered by shorter AP bursts in FHM1 mice. This, together with the enhanced feedforward inhibition mediated by FS INs, may efficiently counteract the increased cortical recurrent excitation. The facilitated recruitment of SOM INs, together with the enhanced recurrent excitation, may contribute to dysfunctional sensory processing in FHM1 and possibly migraine.
